# Supplementary material for: Characteristics and Outcomes of Adult Patients in the PETHEMA Registry with Relapsed or Refractory FLT3-ITD Mutation-Positive Acute Myeloid Leukemia
Source: Cancers (Basel). 2022 Jun 6;14(11):2817. doi: 10.3390/cancers14112817 (PMC9179309; doi:10.3390/cancers14112817)

Supplementary Materials

Supplemental methods

Molecular genetic testing

Molecular genetic testing was performed according to the standard procedures defined in the PETHEMA registry protocols. For detection of FMS-like tyrosine kinase 3 (*FLT3*)-internal tandem duplication (ITD) mutations, DNA was extracted using an automated or manual DNA extraction kit following the manufacturer's instructions. Polymerase chain reaction PCR was conducted using fluorescently labeled primer, followed by capillary electrophoresis using the method of Thiede and colleagues,[1] in order to obtain the mutant/wild type *FLT3*-ITD ratio.

The PETHEMA registry includes an external quality control system among participating laboratories, whereby a biannual/annual exchange of samples is done to ensure the reproducibility of genetic testing results. Furthermore, it is recommended that laboratories participate in external officially recognized quality control programs, such as the United Kingdom National External Quality Assessment Service (UK NEQAS).

---

[1]Thiede, C.; Steudel, C.; Mohr, B.; Schaich, M.; Schakel, U.; Platzbecker, U.; Wermke, M.; Bornhauser, M.; Ritter, M.; Neubauer, A., et al. Analysis of FLT3-activating mutations in 979 patients with acute myelogenous leukemia: association with FAB subtypes and identification of subgroups with poor prognosis. *Blood* **2002**, *99*, 4326-4335.

Supplemental Table S1. The various schedules of salvage (second-line) treatment received by the study population (N=404).

Therapeutic Approach	Schedule	n	Dose
Intensive chemotherapy	IDA + Ara-C (3+7)	42	IDA 10–12 mg/m^2/day D1–3 + Ara-C 100–200 mg/m^2/day D1–7
	HiDAC	6	Ara-C 3000 mg/m^2/12h D1, 3, 5
	Ara-C (intermediate dosing)	6	Ara-C 1000 mg/m^2/day D1–5
	Ara-C + GO	3	Ara-C 100 mg/m^2/day D1–5 + GO 3 mg/m^2/day D1
	IDA + Ara-C (2+5)	2	IDA 10–12 mg/m^2/day D1–2 + Ara-C 100–200 mg/m^2/day D1–5
	DNR + Ara-C (3+7)	7	DNR 60 mg/m^2/day D1–3 + Ara-C 200 mg/m^2/day D1–7
	FLAG-IDA	116	FLU 30 mg/m^2/day D1–4 + IDA 10 mg/m^2/day D1–3 + Ara-C 2000 mg/m^2/day D1–4 + G-CSF 300 µg/ m^2/day D–1–5
	FLAGO-IDA	8	FLU 30 mg/m^2/day D1–4 + IDA 10 mg/m^2/day D1–3 + Ara-C 2000 mg/m^2/day D1–4 + G-CSF 300 µg/m^2/day D–1–5 ± GO 3 mg/m^2/day D1
	FLAT	2	FLU 30 mg/m^2/day + Ara-C 2000 mg/m^2/day + topotecan 1.5 mg/m^2/day D1–4
	MTZ + Ara-C	3	MTZ 10 mg/m^2 + Ara-C 1 g/m^2/12h
	EMA	3	MTZ 8 mg/m^2/day + Ara-C 1000 mg/m^2/day + etoposide 100 mg/m^2/day D1–5
	Allogeneic SCT	6	–
	ICE	1	IDA 10–12 mg/m^2/day D1, 3, 5 + Ara-C 500 mg/m^2/12h D1, 3, 5, 7 + etoposide 100 mg/m^2/day D1–3
	LMA-98	3	IDA 8 mg/m^2/day D1–3 + Ara-C 100 mg/m^2/day D1–7
	MTZ + intermediate Ara-C	1	MTZ 10 mg/m2/day D1–3 + Ara-C 150 mg/m^2/day D1–7
	FLAG	4	FLU 30 mg/m^2/day + Ara-C 1500 mg/m^2/day D1–4 + G-CSF
	Other intensive chemotherapy	47	Amsacrine + Ara-C (n=1), clinical trial (n=4), NA (n=43)
Non-intensive therapy	AZA	25	AZA 75 mg/m^2/day D1–7 or on a 5-on/2-off [weekend]/2-on schedule Q28D
	DEC	5	DEC 20 mg/m^2/daily on days 1–5 q28d
	FLUGA	9	FLU 40 mg/m^2/day orally or 25 mg/m^2/day IV D2–5 or 6 + Ara-C 75 mg/m^2/day D2–5 + G-CSF 5 µg/kg/day D1, 3
	FLAG-IDA-Lite	2	FLU 40 mg/m^2/day D1–5 + Ara-C 20 mg/m^2/day D1–5 + IDA 15 mg/m^2/day D1–3 + G-CSF 300 µg/day D1–5
	Low-dose Ara-C	2	Ara-C, 20 mg/12h D1–10
	Other non-intensive chemotherapy	20	Clinical trial

Supportive care only	Oral treatment<br>No treatment	80	Oral treatment (hydroxyurea, melfalan, etc.)<br>No treatment

Abbreviations: Ara-C, cytarabine; AZA, azacitidine; D, day; DEC, decitabine; DNR, daunorubicin; FLU, fludarabine; G-CSF, granulocyte colony-stimulating factor; GO, gemtuzumab ozogamicin; h, hours; HiDAC, high-dose cytarabine; IDA, idarubicin; IV, intravenous; MTZ, mitoxantrone; NA, not available; Q28D, every 28 days; SCT, stem cell transplantation.

Supplemental Figure S1. First-line active therapies received by the study population (N=404). 3+7, 3 days of daunorubicin plus 7 days of cytarabine. AZA/DEC: azacitidine or decitabine; FLAG: fludarabine, high-dose cytarabine and granulocyte colony-stimulating factor; LDAC: low-dose cytarabine; NA: not available.

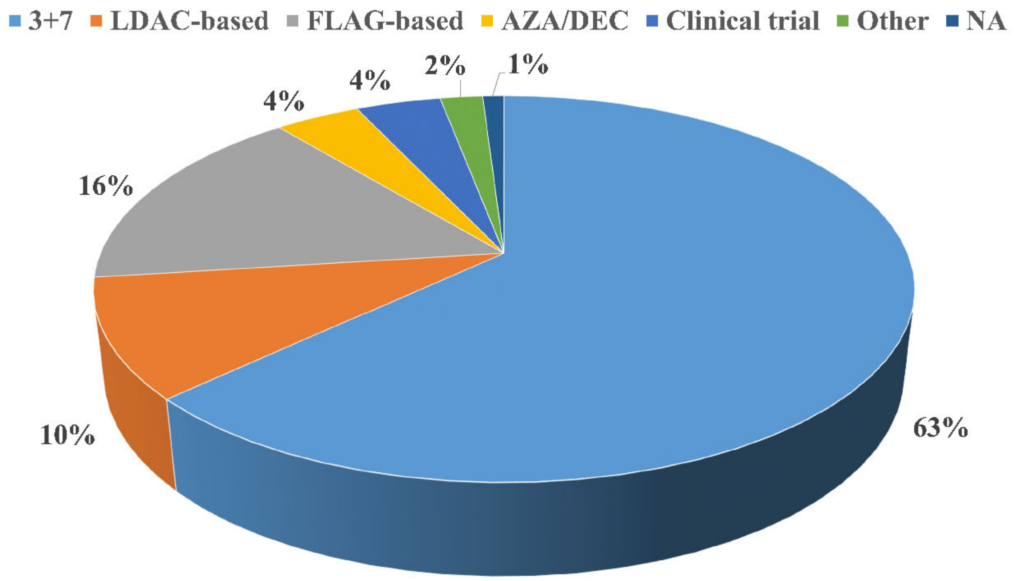

Supplemental Figure S2. Kaplan-Meier plot for overall survival (OS) in the overall cohort.

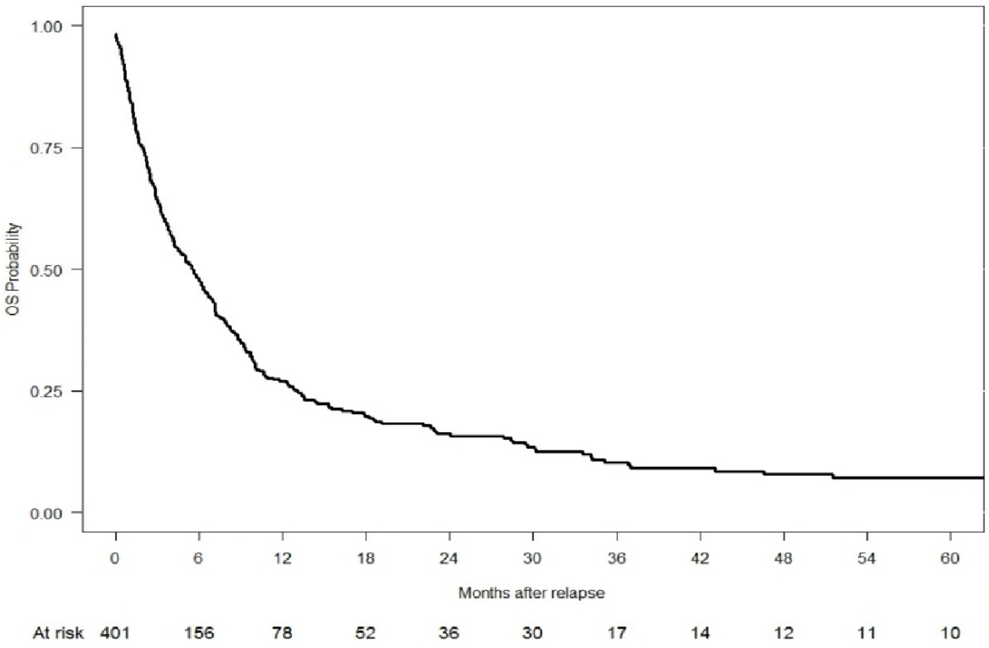

Supplement: Supplementary file 1 [file cancers-14-02817-s001.zip › cancers-1672120-supplementary/Martínez-Cuadrón et al_revised suppl materials_26May22 (clean).pdf]
